# Explainable Multi-Modal Deep Learning for Recording-Level Classification of Respiratory Audio Signals Under Internal and Domain-Shift Evaluation

**DOI:** 10.3390/life16071108

**Published:** 2026-07-02

**Authors:** S M Asiful Islam Saky, Md Saiful Arefin, Md Rashidul Islam, Mohammad Saiful Islam, Rashadul Islam Sumon, Md Mostafizur Rahman Masud, Maria Lapina, Mikhail Babenko, Mohammed Muthanna

**Affiliations:** 1 School of Computing and Informatics, Albukhary International University, Alor Setar 05200, Kedah, Malaysia; saky.aiu22@gmail.com (S.M.A.I.S.); mohammadsaifularefin@gmail.com (M.S.A.); rashidul.md.islam02@gmail.com (M.R.I.); saiful.238161@gmail.com (M.S.I.); 2 Institute of Digital Anti-Aging Healthcare, Inje University, Gimhae 50834, Republic of Korea; sumon39.cst@gmail.com; 3 Faculty of Computing, Universiti Teknologi Malaysia, Johor Bahru 81310, Johor, Malaysia; mrmasud963@gmail.com; 4 Trusted AI Research Center, Russian Academy of Sciences, 109004 Moscow, Russia; mlapina@ncfu.ru (M.L.); mgbabenko@ncfu.ru (M.B.); 5 Department of Computational Mathematics and Cybernetics, North-Caucasus Federal University, 355017 Stavropol, Russia; 6 Faculty of Computing and IT, Sohar University, Sohar 311, Oman

**Keywords:** respiratory sound analysis, recording-level classification, lung disease classification, asthma, COPD, pneumonia, bronchial disease, hybrid deep learning, CNN–BiLSTM, attention mechanism, auscultation, explainable AI, Grad-CAM, integrated gradients, SHAP, probability calibration, reliability curve, external domain-shift evaluation, dataset shift

## Abstract

Respiratory diseases are a major global health challenge. However, identification of respiratory diseases is often limited by subjectivity, environmental noise and inter-clinician variability. This study presents an explainable multimodal deep learning framework for recording-level multiclass classification of respiratory audio signals. The proposed system integrates two complementary representations—a spectro-temporal encoder based on a CNN–BiLSTM-attention architecture and a handcrafted acoustic-feature encoder capturing acoustic descriptors commonly used in respiratory-audio analysis, including MFCCs, zero-crossing rate, spectral centroid, spectral bandwidth, chroma, RMS energy, and spectral rolloff features. These branches are combined through late-stage fusion to leverage both data-driven representation learning and domain-informed acoustic cues. The proposed model was trained and internally evaluated on the Asthma Detection Dataset Version 2, comprising five respiratory categories: bronchial disease, asthma, COPD, healthy, and pneumonia. Mono conversion, resampling to 16 kHz, 100–2000 Hz band-pass filtering, amplitude normalisation, fixed 4 s trimming or zero-padding, training-only augmentation, handcrafted-feature extraction, mel-spectrogram generation, quality control auditing, and stratified recording-level partitioning have been applied in the pre-processing steps. Across five repeated experiments with different random seeds, the proposed hybrid model achieved a mean held-out recording-level test accuracy of 0.9099±0.0163, balanced accuracy of 0.8936±0.0152, macro F1-score of 0.8937±0.0177, macro ROC–AUC of 0.9867±0.0010, and macro PR–AUC of 0.9489±0.0044. Conventional machine learning baseline comparisons showed that the proposed model achieved stronger internal accuracy, balanced accuracy, macro recall, macro F1-score, and macro ROC–AUC than classical machine learning algorithms trained on handcrafted acoustic features, although Random Forest remained competitive in macro PR–AUC. Ablation analysis shows that the deep spectro-temporal branch was the primary contributor to predictive performance, while the handcrafted branch provided complementary interpretable acoustic information rather than consistently improving all classification metrics. Explainability was incorporated using Grad-CAM and Integrated Gradients for spectrogram-based interpretation and SHAP for handcrafted-feature attribution. Domain-shift evaluation on the ICBHI Respiratory Sound Database and a COPD-focused cohort revealed substantial dataset shift effects, including poor healthy-case recognition on ICBHI and seed-dependent COPD recognition in the COPD-focused cohort. Identifier-aware sensitivity analyses showed lower performance than the main recording-level split, suggesting that subject-like or source-level overlap may inflate internal performance estimates. The findings should be interpreted as promising internal held-out recording-level algorithmic performance with limited external transfer, rather than evidence of readiness for clinical use.

## 1. Introduction

Respiratory diseases such as asthma, chronic obstructive pulmonary disease (COPD), bronchial disease, and pneumonia remain major global health challenges contributing substantially to morbidity, mortality, and healthcare burden worldwide [1]. Early identification of respiratory abnormalities is important for timely clinical assessment, appropriate disease management, and reduction of avoidable pressure on healthcare systems. However, access to specialist respiratory assessment may be limited in resource-constrained settings and screening tools that are objective, scalable, and low-cost remain an active area of research.

Pulmonary auscultation is a fundamental component in respiratory assessment, involving listening to lung sounds using a stethoscope. It is non-invasive, inexpensive, and widely used in clinical practice. Nevertheless, its reliability can be affected by clinician experience, inter-observer variability, environmental noise, recording-device differences, and the transient nature of abnormal respiratory sounds [2]. Subtle acoustic events such as wheezes, crackles, airflow irregularities and changes in spectral energy may therefore be missed or interpreted inconsistently. These limitations have motivated research into automated respiratory sound analysis systems that can support more consistent computer-aided auscultation and respiratory screening.

Recent progress in signal processing and artificial intelligence has accelerated the development of machine learning and deep learning methods for respiratory audio classification. Convolutional Neural Networks (CNNs) have been widely used to learn discriminative spectral patterns from time-frequency representations, while recurrent models such as Long Short-Term Memory (LSTM), Bidirectional LSTM (BiLSTM), and Bidirectional Gated Recurrent Units (BiGRUs) have been applied to capture temporal dependencies within respiratory sounds [3,4]. Hybrid CNN–LSTM models combine local spectral feature extraction with temporal sequence modelling and have shown promise in respiratory sound classification tasks [5,6]. Attention mechanisms have also been introduced to emphasise diagnostically informative acoustic segments and improve model transparency [7,8].

Several important challenges remain. Many respiratory audio studies rely primarily on either deep spectrogram-based representations or handcrafted acoustic descriptors, rather than integrating both within a unified multimodal framework. Handcrafted features such as Mel-Frequency Cepstral Coefficients (MFCCs), zero-crossing rate, spectral centroid, spectral bandwidth, chroma, RMS energy, and spectral rolloff provide interpretable acoustic descriptors, whereas deep spectro-temporal models can learn nonlinear discriminative patterns directly from respiratory sound representations. Combining these complementary representations may provide a useful balance between predictive modelling and feature-level interpretability [9]. Second, the interpretability of deep learning models remains a major concern in medical audio applications. Although high classification performance is often reported, the basis of model predictions is not always clear. Explainable AI (XAI) methods such as Grad-CAM, Integrated Gradients, and SHAP can help visualise spectro-temporal attribution and quantify handcrafted-feature contributions, thereby improving transparency in respiratory sound classification [10].

A further challenge is that strong performance on a single dataset or recording-level split does not necessarily imply robust patient-level or cross-dataset generalisation. Respiratory sound datasets may differ in recording devices, sampling rates, patient populations, annotation protocols, disease definitions, background noise, and class distributions. If patient identity is not verified or if duplicate and low-quality recordings are present, recording-level evaluation may overestimate real-world performance. Therefore, respiratory audio models should be interpreted cautiously unless supported by quality control auditing, repeated-seed evaluation, sensitivity analysis, and external domain-shift evaluation under clearly described label-mapping assumptions.

This study proposes an explainable hybrid deep learning framework for recording-level multiclass respiratory audio classification. The proposed architecture integrates a CNN-BiLSTM-attention network for deep spectro-temporal representation learning with a parallel handcrafted-feature encoder that captures interpretable acoustic descriptors. These complementary streams are fused using a late-stage fusion strategy to combine data-driven respiratory sound representations with domain-informed acoustic cues. Grad-CAM and Integrated Gradients are used for spectrogram-based interpretation, while SHAP is applied to handcrafted features for feature-level attribution in order to improve transparency. In response to concerns about reproducibility and generalisability, this study also reports quality control auditing, repeated-seed evaluation, baseline comparisons, ablation analysis, probability-based discrimination and calibration metrics, and external domain-shift evaluation. The domain-shift evaluation uses the ICBHI Respiratory Sound Database and a COPD-focused respiratory dataset without retraining or fine-tuning. Because the external datasets do not share identical label definitions with the original training dataset, these analyses are treated as domain-shift evaluations rather than evidence of readiness for clinical use. This framing allows the study to assess both the promise and the limitations of the proposed approach under more realistic cross-dataset conditions.

The remainder of this paper is organised as follows. Section 2 reviews recent advances in deep learning, multimodal fusion, temporal modelling, and XAI for respiratory sound analysis. Section 3 details the proposed framework, including data acquisition, quality control auditing, preprocessing, feature extraction, model architecture, training strategy, evaluation protocol, baseline comparison, ablation analysis, and external domain-shift evaluation design. Section 4 presents the experimental results, including internal held-out recording-level performance, repeated-seed analysis, baseline comparison, ablation findings, external-domain-shift evaluation, and interpretability analysis. Finally, Section 5 concludes the study and outlines future research directions.

## 2. Literature Review

### 2.1. Deep Learning in Respiratory Sound Classification

Deep learning has become an important approach for respiratory sound analysis because lung sounds contain complex time-frequency patterns that are difficult to characterise reliably using manual auscultation alone. These acoustic events can be intermittent, subtle, and affected by recording noise, patient breathing pattern, device characteristics, and clinician experience. Automated respiratory sound analysis therefore offers a potential route toward more consistent computer-aided auscultation, although its reliability depends strongly on dataset quality, evaluation protocol, and cross-dataset robustness.

Convolutional Neural Networks (CNNs) have been widely used for respiratory sound classification because they can learn local discriminative patterns from time-frequency representations such as mel-spectrograms, MFCC maps, and other spectral images. Joy and Haider [3] used CNN-based models for respiratory disorder classification from acoustic features, while Kim et al. [4] investigated CNN-based abnormal respiratory sound recognition, including crackle and wheeze identification. Vasava and Joshiara [11] further demonstrated the utility of converting respiratory audio into two-dimensional spectrogram representations for deep convolutional analysis. Other studies have explored architectural improvements such as depthwise separable convolutions, residual blocks, and attention-enhanced CNNs to improve feature extraction from noisy respiratory recordings [12,13]. These studies support the usefulness of CNNs as spectral feature extractors for lung sound analysis.

However, CNN-only models may not fully capture the temporal structure of respiratory sounds, particularly when abnormal events occur at specific points within inspiration or expiration. To address this limitation, recurrent and hybrid deep learning architectures have been introduced. LSTM, BiLSTM, and BiGRU models can capture sequential dependencies across time and are therefore suitable for modelling respiratory cycles and intermittent pathological events. Hybrid CNN-LSTM models combine CNN-based spectral feature extraction with recurrent temporal modelling and have shown promise in respiratory sound classification tasks [5,6]. Petmezas et al. [14] and related works have also explored multi-branch or recurrent-enhanced models to improve temporal representation and classification robustness [15,16]. These methods demonstrate that combining spectral and temporal modelling can be beneficial, but their reported performance often remains closely tied to the dataset and evaluation protocol used.

A common limitation in this literature is that many studies report strong within-dataset performance but provide limited evidence of generalisation across independent datasets, recording devices, or patient populations. Differences in sampling rate, recording duration, microphone or stethoscope type, anatomical recording location, annotation protocol, and disease-label definition can substantially affect model behaviour. As a result, high accuracy on a single dataset, especially under recording-level partitioning, may not reflect real-world robustness. This limitation is particularly important in public respiratory sound datasets, where verified patient identifiers, duplicate recordings, and recording quality issues may not always be fully controlled.

### 2.2. Hybrid and Multimodal Feature Fusion Approaches

Respiratory audio can be represented using both handcrafted acoustic descriptors and deep-learned spectro-temporal embeddings. Handcrafted features, including MFCCs, zero-crossing rate, spectral centroid, spectral bandwidth, chroma, RMS energy, and spectral rolloff, provide compact and interpretable summaries of the respiratory signal. These descriptors can reflect spectral envelope, airflow turbulence, harmonic structure, signal energy, and frequency distribution. In contrast, deep representations learned from mel-spectrograms can capture nonlinear and localised acoustic patterns that may not be fully described by handcrafted statistics.

Several recent studies have investigated multimodal or hybrid feature-fusion strategies to combine these complementary representations. Karaarslan et al. [9] used statistical sound features with feature-selection strategies for respiratory classification, showing the continued relevance of domain-informed acoustic descriptors. Gopi and Varghese [7] proposed a multi-input deep learning architecture that combines different feature representations, including MFCCs, chroma features, and mel-spectrograms. Shehab et al. [10] and Singh et al. [17] also explored deep-feature-level fusion across multiple neural encoders for respiratory sound analysis. Other multimodal frameworks have investigated cross-representation alignment and attention-based fusion to improve discriminative modelling under heterogeneous acoustic conditions [18,19]. Mixture-of-experts and multi-channel methods have similarly been used to exploit channel-specific or representation-specific specialisation [20].

Although these works show that hybrid and multimodal approaches can improve representation richness, the benefit of handcrafted-feature fusion is not always consistent across datasets or metrics. In some cases, the deep spectro-temporal branch may dominate predictive performance, while handcrafted features contribute more clearly to interpretability than to absolute accuracy. Therefore, hybrid fusion should not be assumed to be universally superior. Instead, its value should be evaluated through ablation analysis, repeated-seed testing, probability-based discrimination analysis, and external domain-shift evaluation. This distinction is important for the present study, where the handcrafted branch is treated as a complementary and interpretable acoustic representation rather than as a guaranteed performance-improving component.

### 2.3. Attention Mechanisms and Temporal Modelling

Attention mechanisms are increasingly used in respiratory sound classification to identify acoustically informative regions within time-frequency representations or temporal feature sequences. Pathological respiratory sounds are often localised in time and frequency. Wheezes may appear as narrowband tonal patterns, while crackles may appear as short broadband bursts. Attention modules can help the model emphasise these diagnostically relevant segments instead of weighting all frames equally.

Earlier hybrid temporal models such as CNN–BiGRU and CNN–LSTM frameworks demonstrated the importance of sequential modelling in respiratory sound classification [20,21,22]. More recent approaches have incorporated attention mechanisms to dynamically weight salient respiratory segments. Sanjana et al. [8] introduced attention-based CRNN architectures for multiclass respiratory disease identification, while self-attention and temporal gating methods have been used to improve the localisation of acoustic abnormalities in continuous recordings [23,24]. Temporal Convolutional Networks have also been explored as an alternative approach for modelling respiratory events over time [25]. These studies suggest that temporal modelling and attention can improve the ability of deep models to capture non-stationary respiratory patterns.

However, attention weights should not be interpreted automatically as clinical explanations. Attention mechanisms may highlight discriminative acoustic regions, but these regions require careful validation to determine whether they correspond to clinically meaningful respiratory events rather than dataset-specific artifacts, recording noise, or device-dependent patterns. Therefore, attention-based models should ideally be paired with additional interpretability tools and, where possible, expert review of explanation outputs.

### 2.4. Explainable AI in Medical Audio Diagnosis

Explainable AI (XAI) is particularly important in medical audio applications because high predictive performance alone is insufficient for clinical trust or responsible use. In respiratory sound analysis, explanation methods can help identify which parts of a spectrogram or which acoustic descriptors contributed to a model decision. Grad-CAM can localise discriminative spectro-temporal regions in convolutional feature maps, Integrated Gradients can estimate attribution across input spectrogram bins, and SHAP can quantify the contribution of handcrafted acoustic features [26]. Prior respiratory audio studies have used XAI to relate model attention to acoustic phenomena such as wheezes and crackles [27]. Broader healthcare XAI research has also emphasised the importance of transparency, accountability, and cautious interpretation in high-risk medical contexts [28,29]. Systematic reviews further indicate that explainability is an important requirement for respiratory audio diagnosis, but that explanation quality and clinical validation remain open challenges [2]. Despite its usefulness, post-hoc XAI has several limitations. XAI results should be interpreted as model behaviour analysis rather than clinical validation unless supported by expert assessment.

### 2.5. Research Gap and Positioning of the Present Study

The reviewed literature shows that deep learning, temporal modelling, attention mechanisms, multimodal fusion, and XAI have all contributed to progress in automated respiratory sound classification. Nevertheless, several gaps remain. First, many studies emphasise performance on a single dataset and provide limited analysis of duplicate recordings, clipping, recording-quality issues, patient-level separation, or external dataset shift. Second, hybrid models often claim improved performance from feature fusion without fully disentangling the contributions of the deep and handcrafted branches. Third, XAI is frequently presented qualitatively, with limited discussion of its limitations or lack of clinical validation. Fourth, domain-shift evaluation across datasets with different label definitions and acquisition protocols remains underreported.

The present study addresses these gaps by proposing an explainable hybrid CNN–BiLSTM–attention model that combines mel-spectrogram-based spectro-temporal learning with a handcrafted acoustic-feature encoder. The contribution is not limited to the model architecture; the revised experimental design also includes quality control auditing, repeated-seed evaluation, baseline comparison, ablation analysis, probability-based discrimination and calibration metrics, and external domain-shift evaluation. Importantly, the study interprets the external domain-shift evaluation results conservatively. Rather than treating external testing as evidence of readiness for clinical use, the domain-shift evaluation is used to assess the limits of cross-dataset transfer and to identify where future domain adaptation, harmonised labels, verified patient-level evaluation, and prospective validation are needed.

## 3. Methodology

### 3.1. Overview of the Proposed Framework

**Figure 1:**
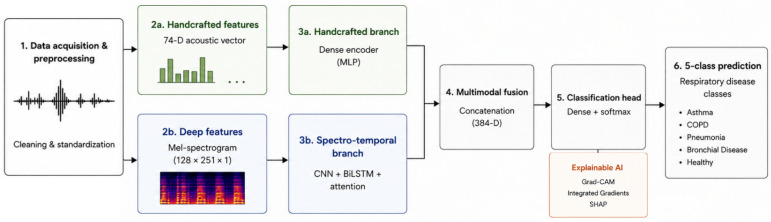
Overview of the proposed explainable hybrid multimodal CNN–BiLSTM–attention framework for respiratory audio classification. The framework includes audio preprocessing, mel-spectrogram generation, handcrafted acoustic-feature extraction, late-stage multimodal fusion, softmax classification, and post hoc interpretability using Grad-CAM, Integrated Gradients, and SHAP.

The proposed framework illustrated in Figure 1 is a multimodal deep learning pipeline for recording-level multiclass respiratory audio classification. The system first applies a standardised preprocessing pipeline to each audio recording, including mono conversion when required, resampling, band-pass filtering, amplitude normalisation, and fixed-duration trimming or zero-padding. Two complementary feature representations are then extracted. The first branch converts the processed waveform into a mel-spectrogram and uses a CNN–BiLSTM–attention network to learn deep spectro-temporal patterns. The second branch extracts handcrafted acoustic descriptors, including MFCCs, zero-crossing rate, spectral centroid, spectral bandwidth, chroma, RMS energy, and spectral rolloff features. These handcrafted descriptors are processed through a dense encoder and fused with deep spectro-temporal embedding using a late-fusion strategy.

The final fused representation is passed to a softmax classifier to predict one of five respiratory categories: Asthma, Bronchial, COPD, Healthy, or Pneumonia. Grad-CAM and Integrated Gradients are applied to the mel-spectrogram branch to improve model transparency, while SHAP is applied to the handcrafted-feature branch. These methods are used to analyze model behaviour and feature attribution. However, they are not interpreted as clinical validation without expert confirmation.

### 3.2. Data Acquisition

The internal development and evaluation experiments were conducted using the publicly available Asthma Detection Dataset Version 2 [30]. The dataset contains respiratory audio recordings from five categories—Asthma, Bronchial, COPD, Healthy, and Pneumonia. A total of 1211 WAV recordings were used in the main experimental pipeline. The class-wise distribution is shown in Table 1. The dataset is imbalanced, with COPD representing the largest class and Bronchial representing the smallest class. Therefore, macro-averaged metrics, balanced accuracy, ROC–AUC, and PR–AUC were included to provide a more reliable evaluation under class imbalance.

A file-level audit confirmed that all 1211 recordings were stored in WAV format and were single-channel mono recordings. The original sampling rates were heterogeneous, with 707 recordings sampled at 44.1 kHz and 504 recordings sampled at 4 kHz. Recording duration ranged from 0.48 s to 6.00 s. Because of these differences, all files were resampled and temporally standardised before feature extraction. Exploratory data analysis was performed to examine class distribution, recording duration, sampling-rate variation, amplitude characteristics, clipping, duplicate files, and spectro-temporal patterns. This audit informed the preprocessing, quality control reporting, feature extraction, and evaluation strategy used in this study.

### 3.3. Data Preprocessing

**Figure 2:**
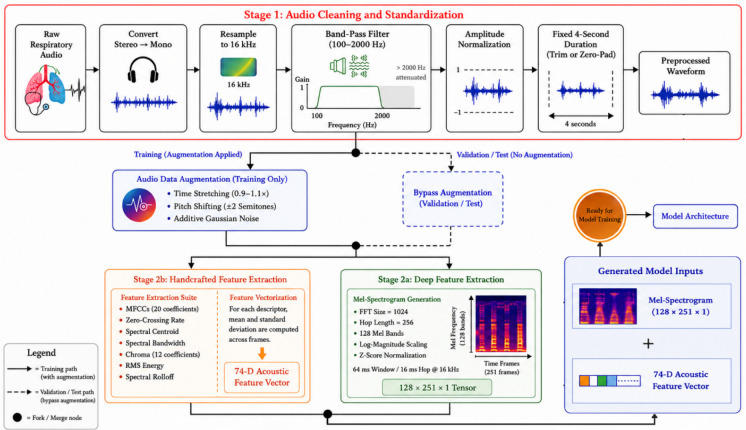
Final preprocessing and multimodal feature-extraction pipeline. The workflow includes mono conversion, resampling to 16 kHz, 100–2000 Hz band-pass filtering, amplitude normalisation, fixed 4 s trimming or zero-padding, training-only augmentation, handcrafted acoustic-feature extraction, mel-spectrogram generation, and recording-level data partitioning.

All respiratory recordings were processed using a consistent preprocessing pipeline designed to standardise the input format while preserving diagnostically relevant respiratory acoustic information. As shown in Figure 2, the pipeline included mono conversion when required, resampling to 16 kHz, 100–2000 Hz band-pass filtering, amplitude normalisation, waveform standardisation, and fixed 4 s trimming or zero-padding. Data augmentation was applied only during training and was not applied to validation, held-out test, or external domain-shift recordings.

#### 3.3.1. Audio Resampling and Temporal Standardisation

All recordings were resampled to 16 kHz to ensure a uniform processing rate across files originally recorded at different sampling frequencies. However, resampling does not recover frequency components absent from lower-sampling-rate recordings. Therefore, the effective analysable bandwidth was constrained by both the original acquisition rate and the subsequent filtering step. In the final implementation, a 100–2000 Hz band-pass filter was applied to focus the analysis on the respiratory sound frequency range used by the model and to reduce low-frequency drift and high-frequency noise.

Each recording was standardised to a fixed duration of 4 s, corresponding to 64,000 samples at 16 kHz. Recordings longer than 4 s were trimmed, while recordings shorter than 4 s were zero-padded. After filtering and temporal standardisation, each waveform was amplitude-normalised and standardised to reduce scale variability and improve numerical stability during model training.

#### 3.3.2. Quality Control Audit and Noise-Robust Processing

A quality control audit was conducted prior to model development to document potential technical issues in the dataset. The audit examined loading errors, number of channels, original sampling rates, duration range, very short recordings, recordings shorter than the model input length, near-silent recordings, low-peak recordings, excessive clipping, and exact duplicate audio files. The purpose of this audit was to document dataset quality and identify potential sources of optimistic performance estimation, not to remove files from the main experimental pipeline.

All 1211 recordings were retained in the main analysis to preserve the original public dataset composition. However, as shown in Table 2, the audit identified 171 recordings with clipped samples exceeding 1%, including 127 recordings in the training set, 21 in the validation set, and 23 in the held-out test set. The audit also identified 12 exact duplicate audio pairs. These characteristics are important because clipped or duplicated recordings may inflate apparent performance if distributed across different data partitions. Therefore, the revised study reports these issues transparently and interprets the main recording-level results cautiously.

#### 3.3.3. Data Augmentation

The probabilistic augmentation was applied during training only to reduce overfitting and improve robustness to acoustic variability that including time stretching, pitch shifting, and additive Gaussian noise. Time stretching randomly scaled the audio waveform by a factor between 0.9 and 1.1, simulating natural variation in breathing rate. Pitch shifting modified the signal by up to ±2 semitones, reflecting speaker-related and device-related acoustic differences. Additive Gaussian noise was used to simulate mild background disturbance. These transformations were applied only to training samples and were not applied to validation, held-out test, or external domain-shift samples.

#### 3.3.4. Dataset Partitioning

The internal dataset was partitioned into training, validation, and held-out test subsets using a stratified recording-level split. The split followed an approximately 70/15/15 ratio, resulting in 847 training recordings, 182 validation recordings, and 182 held-out test recordings. The class-wise distribution of the training, validation, and held-out test subsets is reported in Table 3. The training set was used for model optimisation, the validation set was used for model selection and early stopping, and the held-out test set was used only for final internal recording-level evaluation. A fixed random seed was used to improve reproducibility.

Although filenames contain leading subject-like identifiers such as P1, P2, and P10, these identifiers are reused across disease folders and could not be verified as globally unique patient identifiers. Therefore, patient-level separation could not be confirmed. The main internal evaluation is consequently reported as a stratified recording-level evaluation rather than a verified patient-level evaluation. This limitation is explicitly acknowledged when interpreting the results.

### 3.4. Feature Extraction

Feature extraction was performed using a dual-representation strategy. The deep-learning branch used mel-spectrograms to capture spectro-temporal patterns, while the handcrafted branch used acoustic descriptors to provide interpretable statistical summaries of the respiratory signal. Both representations were extracted after the same preprocessing pipeline.

#### 3.4.1. Mel-Spectrogram Features

Each preprocessed 4 s waveform was converted into a 128-band mel-spectrogram using the Short-Time Fourier Transform (STFT). The STFT of a discrete-time signal x[n] is defined as

(1) $$\mathrm{STFT}\left\{x\left[n\right]\right\}\left(t,f\right)=\sum_{n=0}^{N-1}x\left[n\right]\;w\left[n-t\right]\;e^{-j2\pi fn/N},$$

where w[·] denotes the analysis window and *N* denotes the frame length. In the final implementation, an FFT size of 1024 samples and a hop length of 256 samples were used at a sampling rate of 16 kHz. The resulting magnitude spectrogram was projected onto 128 mel bands, converted to the decibel domain, and normalised using z-score standardisation. The final mel-spectrogram input shape was 128×251×1.

#### 3.4.2. Handcrafted Acoustic Features

A 74-dimensional handcrafted acoustic feature vector was extracted from each preprocessed waveform using Librosa to complement the deep spectro-temporal branch. The feature set included 20 MFCCs, zero-crossing rate, spectral centroid, spectral bandwidth, 12 chroma coefficients, RMS energy, and spectral rolloff. For each descriptor group, the mean and standard deviation were computed across frames. This produced 40 MFCC features, two zero-crossing-rate features, two spectral-centroid features, two spectral-bandwidth features, 24 chroma features, two RMS-energy features, and two spectral-rolloff features, resulting in 74 handcrafted features in total.

(2) $$f_{\mathrm{hand}}=\left[\mu_{\mathrm{MFCC}},\sigma_{\mathrm{MFCC}},\mu_{\mathrm{ZCR}},\sigma_{\mathrm{ZCR}},\mu_{\mathrm{Centroid}},\sigma_{\mathrm{Centroid}},\mu_{\mathrm{Bandwidth}},\sigma_{\mathrm{Bandwidth}},\mu_{\mathrm{Chroma}},\sigma_{\mathrm{Chroma}},\mu_{\mathrm{RMS}},\sigma_{\mathrm{RMS}},\mu_{\mathrm{Rolloff}},\sigma_{\mathrm{Rolloff}}\right]\in R^{74}.$$

These descriptors were included to provide interpretable acoustic information related to spectral envelope, airflow irregularity, frequency distribution, harmonic structure, signal energy, and spectral rolloff behaviour.

#### 3.4.3. Hybrid Feature Representation

The proposed system combines the mel-spectrogram representation and handcrafted acoustic representation through a dual-branch architecture. The CNN–BiLSTM–attention branch processes the mel-spectrogram and produces a deep spectro-temporal embedding. The handcrafted-feature branch transforms the 74-dimensional acoustic descriptor vector into a lower-dimensional latent representation. The two embeddings are fused by concatenation:

(3) $$f_{\mathrm{fusion}}=\left[f_{\mathrm{deep}}\|f_{\mathrm{hand}}\right],$$

where ‖ denotes vector concatenation. Late fusion was selected to preserve branch-specific representations before classification. In the revised interpretation, the handcrafted branch is treated primarily as a complementary and interpretable acoustic representation rather than as a component that consistently improves every performance metric.

### 3.5. Hybrid Model Architecture

**Figure 3:**
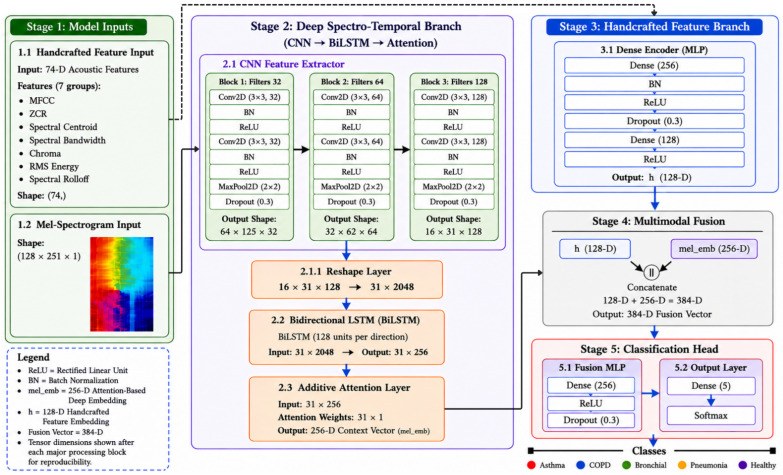
Overview of the proposed hybrid multimodal CNN–BiLSTM–attention architecture. The mel-spectrogram input has shape 128×251×1, and the handcrafted acoustic-feature input has dimension 74.

The proposed architecture integrates a deep spectro-temporal learning pathway and a handcrafted acoustic-feature pathway, as shown in Figure 3. The deep pathway learns from mel-spectrograms using CNN, BiLSTM, and additive attention layers. The handcrafted pathway encodes the 74-dimensional acoustic descriptor vector using dense layers. The two representations are concatenated and passed through a fusion classifier.

#### 3.5.1. CNN-Based Spectrogram Encoder

Each respiratory signal is represented as a mel-spectrogram $S\in R^{128\times 251\times 1}$. The spectrogram is processed using a hierarchical convolutional encoder composed of convolution, batch normalisation, ReLU activation, max pooling, and dropout operations:

(4) Conv2D(k×k,f)→BatchNorm→ReLU→MaxPooling→Dropout,

where *k* is the kernel size and *f* is the number of filters. This encoder learns local spectro-temporal features from the respiratory audio representation.

#### 3.5.2. BiLSTM-Based Temporal Modelling

The convolutional feature maps are reshaped into a temporal sequence and passed to a Bidirectional Long Short-Term Memory network. The BiLSTM processes the sequence in both forward and backward directions:

(5) $${\vec{h}}_{t}=\mathrm{LSTM}\left(F_{c,t}\right),\;{\overset{\leftarrow }{h}}_{t}=\mathrm{LSTM}\left(F_{c,T-t+1}\right),\;h_{t}=\left[{\vec{h}}_{t};{\overset{\leftarrow }{h}}_{t}\right],$$

where $F_{c,t}$ denotes the convolutional feature representation at time step *t*. This bidirectional temporal modelling allows the network to capture sequential dependencies across the respiratory sound representation.

#### 3.5.3. Additive Attention Mechanism

An additive attention mechanism is applied to the BiLSTM outputs to assign different importance weights to different temporal frames. The attention score $e_{t}$, normalised attention weight $\alpha_{t}$, and context vector *c* are defined as

(6) $$e_{t}=v^{⊤}\tanh\left(Wh_{t}+b\right),\;\alpha_{t}=\frac{\exp(e_{t})}{\sum_{i=1}^{T^{\prime }}\exp\left(e_{i}\right)},\;c=\sum_{t=1}^{T^{\prime }}\alpha_{t}h_{t}.$$

The resulting context vector summarises the temporal information emphasised by the attention layer. Attention is used as a model component for temporal weighting; however, attention weights alone are not treated as clinical explanations without further validation.

#### 3.5.4. Handcrafted-Feature Encoder

In parallel, the handcrafted input vector $x_{h}\in R^{74}$ is passed through a lightweight feed-forward encoder. This encoder applies dense transformations, batch normalisation, ReLU activation, and dropout:

(7) $$x_{h1}=\mathrm{ReLU}\left(\mathrm{BN}\left(W_{1}x_{h}+b_{1}\right)\right),\;x_{h2}={\mathrm{Dropout}}_{0.3}\left(\mathrm{ReLU}(\mathrm{BN}\left(W_{2}x_{h1}+b_{2}\right))\right).$$

This branch provides a compact latent representation of interpretable acoustic descriptors.

#### 3.5.5. Feature Fusion and Classification

The deep spectro-temporal embedding $E_{m}$ and the handcrafted embedding $E_{h}$ are concatenated to form the fused multimodal representation:

(8) $$z=[E_{m};E_{h}].$$

The fused representation is passed through a dense fusion layer followed by dropout and a softmax classifier:

(9) $$\hat{y}=\mathrm{Softmax}\left(W_{f}z+b_{f}\right),$$

where $\hat{y}$ denotes the predicted probability distribution over the five classes: Asthma, Bronchial, COPD, Healthy, and Pneumonia. The final architecture supports post hoc interpretation using Grad-CAM and Integrated Gradients on the spectrogram branch and SHAP on the handcrafted-feature branch.

### 3.6. Training Strategy

The proposed hybrid model was trained using a controlled optimisation protocol designed to support stable convergence and reproducibility. The final configuration included AdamW optimisation, learning-rate scheduling, dropout regularisation, early stopping, model checkpointing, and repeated-seed evaluation.

#### 3.6.1. Optimisation and Hyperparameter Configuration

Model optimisation was performed using the AdamW optimiser with an initial learning rate of $\eta_{0}=3\times 10^{-4}$ and weight decay of $1\times 10^{-5}$. A ReduceLROnPlateau scheduler monitored validation loss and reduced the learning rate by a factor of 0.5 with a patience of 6 epochs and a minimum learning rate of $1\times 10^{-6}$. The maximum number of epochs was 100, and early stopping with patience 12 was used to reduce overfitting. The batch size was 16.

#### 3.6.2. Loss Function and Regularisation

The model was optimised using Sparse Categorical Cross-Entropy with label smoothing. The smoothed objective is defined as

(10) $$L_{\mathrm{SCCE}\text{-}\mathrm{smoothed}}=-\frac{1}{N}\sum_{i=1}^{N}\sum_{c=1}^{C}\left[\left(1-\epsilon \right)y_{i,c}+\frac{\epsilon }{C}\right]\log\left({\hat{y}}_{i,c}\right),$$

where *N* is the batch size, *C* is the number of classes, $y_{i,c}$ is the one-hot class label, ${\hat{y}}_{i,c}$ is the predicted probability, and ϵ=0.05 is the label-smoothing factor. Dropout rates between 0.3 and 0.4 and AdamW weight decay were used for regularisation.

#### 3.6.3. Training Protocol and Class-Imbalance Handling

Training progress was monitored using validation loss and validation performance. The best checkpoint was selected based on validation performance, while the held-out recording-level test set was kept isolated until final internal evaluation. The final main deep-learning experiment did not apply class weighting, preserving the original training behaviour. Macro precision, macro recall, macro F1-score, balanced accuracy, macro ROC–AUC, and macro PR–AUC were reported to account for class imbalance during evaluation. Classical machine learning baselines were trained with balanced class weighting where applicable. The Full Hybrid model was trained across five random seeds to ass stability: 42, 101, 202, 303, and 404. Results were summarised using mean ± standard deviation.

#### 3.6.4. Summary of Training Configuration

The final optimisation settings, regularisation choices, data split, repeated-run seeds, and evaluation metrics used for model training are summarised in Table 4.

### 3.7. Baseline Comparison Protocol

Conventional machine learning baselines were trained using the same internal training and held-out test partitions to evaluate the incremental value of the proposed hybrid model. Logistic regression, support vector machine with radial basis function kernel (SVM-RBF), and Random Forest classifiers were trained on handcrafted acoustic features. Standardisation was applied where required, and balanced class weighting was used for applicable classical models. These baselines were evaluated using the same internal held-out recording-level metrics as the proposed model. In addition to conventional machine learning baselines, deep learning ablation models were used to assess the contribution of the main architectural components. The baseline and ablation experiments were interpreted as internal comparisons rather than evidence of external clinical generalisation.

### 3.8. Ablation Study

An ablation study was conducted to evaluate the contribution of the main architectural components. Each variant used the same preprocessing pipeline, internal dataset split, and general training protocol.

1. Full Hybrid Model: The complete proposed architecture, including the CNN spectrogram encoder, BiLSTM temporal modelling, additive attention, handcrafted-feature encoder, and late-stage fusion.
2. Deep-Only Model: This variant removes the handcrafted-feature branch and uses only the mel-spectrogram-based CNN–BiLSTM–attention pathway.
3. Handcrafted-Only Model: This variant removes the mel-spectrogram branch and uses only handcrafted acoustic descriptors with a dense classifier.
4. CNN-Only Model: This variant removes the BiLSTM and attention components from the deep branch and uses convolutional feature aggregation before classification.
5. No-Attention Model: This variant retains the CNN and BiLSTM components but replaces the additive attention mechanism with simpler temporal aggregation.

The ablation analysis was used to understand the architectural contribution rather than to claim the universal superiority of the Full Hybrid model. In the revised interpretation, the Full Hybrid model is treated as providing competitive internal performance with additional handcrafted-feature interpretability, while the deep spectro-temporal branch is recognised as the primary contributor to predictive performance.

### 3.9. Evaluation and Validation

#### 3.9.1. Evaluation Metrics

Multiple metrics were used to evaluate classification performance. Accuracy was used to summarise overall correctness, while balanced accuracy and macro-averaged metrics were emphasised because of class imbalance. For a given class, let TP, TN, FP, and FN denote true positive, true negative, false positive, and false negative counts. Standard metrics were computed as follows:

(11) $$\mathrm{Accuracy}=\frac{TP+TN}{TP+TN+FP+FN},$$

(12) $$\mathrm{Precision}=\frac{TP}{TP+FP},$$

(13) $$\mathrm{Recall}=\frac{TP}{TP+FN},$$

(14) $$F\text{-}\mathrm{Score}=2\cdot \frac{\mathrm{Precision}\cdot \mathrm{Recall}}{\mathrm{Precision}+\mathrm{Recall}}.$$

Balanced accuracy was computed as the average recall across classes. Macro ROC–AUC and macro PR–AUC were used to assess threshold-independent discriminative performance, with PR–AUC included because it is informative under class imbalance. Because ROC–AUC, PR–AUC, and softmax scores assess discrimination rather than the reliability of predicted probabilities, probability calibration was additionally evaluated using expected calibration error (ECE), Brier score, negative log likelihood (NLL), and reliability curves. These calibration analyses were used to characterise internal model behaviour and were not interpreted as evidence that predicted probabilities are clinically actionable.

#### 3.9.2. Internal Validation and Reproducibility Protocol

The validation and held-out test subsets were derived from the stratified recording-level partitioning strategy described in Section 3.3.4. The validation set was used for model selection, learning-rate scheduling, and early stopping. The held-out test set was used only for final internal recording-level assessment.

Confusion matrices, classification reports, ROC curves, precision–recall curves, repeated-seed summaries, baseline comparisons, and ablation results were generated to characterise internal model behaviour. Because verified patient-level separation could not be guaranteed, the internal test results are reported as held-out recording-level results rather than patient-level performance.

### 3.10. External Domain-Shift Evaluation

External domain-shift evaluation was added to evaluate cross-dataset transfer without retraining or fine-tuning. The five repeated-seed Full Hybrid models were evaluated on two external respiratory audio resources: the ICBHI Respiratory Sound Database [31] and RespiratoryDatabase@TR [32].

The ICBHI database was used as the main external cohort. Because its diagnostic labels are not fully equivalent to the five labels used in the internal training dataset, two evaluation settings were defined. The primary external setting was Healthy versus Disease classification, where ICBHI Healthy recordings were treated as Healthy and all other ICBHI diagnostic categories were treated as Disease. The secondary setting was an overlapping four-class analysis using Asthma, COPD, Healthy, and Pneumonia. ICBHI recordings were divided into fixed 4 s windows to match the trained model input duration. Window-level probabilities were averaged to recording level and then to patient level.

RespiratoryDatabase@TR was used as a secondary COPD-focused external cohort. Because this dataset provides COPD severity labels rather than the same five diagnostic categories used in the internal training dataset, it was not treated as full five-class external domain-shift evaluation. Instead, the analysis focused on COPD prediction rate, mean COPD probability, prediction distribution, and the association between predicted COPD probability and COPD severity stage using Spearman correlation and Kruskal–Wallis testing.

These external analyses were designed to assess domain-shift behaviour rather than to demonstrate readiness for clinical use. Differences in recording protocol, sampling characteristics, disease-label definitions, patient population, and class distribution were considered when interpreting the external results.

### 3.11. Explainable AI Analysis

The proposed framework incorporates Grad-CAM, Integrated Gradients, and SHAP to improve transparency. Grad-CAM and Integrated Gradients were applied to the mel-spectrogram branch to examine spectro-temporal regions contributing to model predictions. SHAP was applied to the handcrafted-feature branch to estimate the contribution of acoustic descriptors such as MFCCs, zero-crossing rate, spectral centroid, spectral bandwidth, chroma, RMS energy, and spectral rolloff.

#### 3.11.1. Grad-CAM

Grad-CAM was used to visualise class-discriminative spectro-temporal regions in the convolutional feature maps. The importance weight $\alpha_{k}$ for feature map $A_{k}$ is computed as

(15) $$\alpha_{k}=\frac{1}{Z}\sum_{i}\sum_{j}\frac{\partial y_{c}}{\partial A_{ij}^{k}},$$

where $y_{c}$ is the target class score and *Z* is the number of spatial locations. The class activation map is then computed as

(16) $$L_{c}^{\mathrm{Grad}\text{-}\mathrm{CAM}}=\mathrm{ReLU}\left(\sum_{k}\alpha_{k}A_{k}\right).$$

The resulting heatmaps were superimposed on mel-spectrograms to identify regions that influenced the model prediction.

#### 3.11.2. Integrated Gradients

Integrated Gradients was used to estimate attribution across the mel-spectrogram input. Given an input *x* and baseline $x^{\prime }$, the attribution for input feature $x_{i}$ is defined as

(17) $$IG_{i}\left(x\right)=\left(x_{i}-x_{i}^{\prime }\right)\int_{0}^{1}\frac{\partial F(x^{\prime }+\alpha \left(x-x^{\prime }\right))}{\partial x_{i}}d\alpha ,$$

where F(·) denotes the model output. A zero-valued spectrogram was used as the baseline.

#### 3.11.3. SHAP Analysis

SHAP was applied to the handcrafted-feature branch to estimate feature-level contributions. The SHAP value $\phi_{i}$ for feature $x_{i}$ is defined as

(18) $$\phi_{i}=\sum_{S\subseteq N\setminus \{i\}}\frac{|S|!(|N|-|S|-1)!}{N!}\left[f(S\cup \{i\})-f(S)\right],$$

where *S* is a subset of features, *N* is the full feature set, and f(·) is the model prediction function.

The XAI outputs were used to analyse model behaviour and provide feature-level transparency. However, the explanations were not independently validated by clinical experts. Therefore, the XAI results should be interpreted as post hoc model-behaviour analysis rather than clinical evidence that the model relies on validated respiratory biomarkers.

## 4. Results and Discussion

### 4.1. Learning Curves and Model Convergence

**Figure 4:**
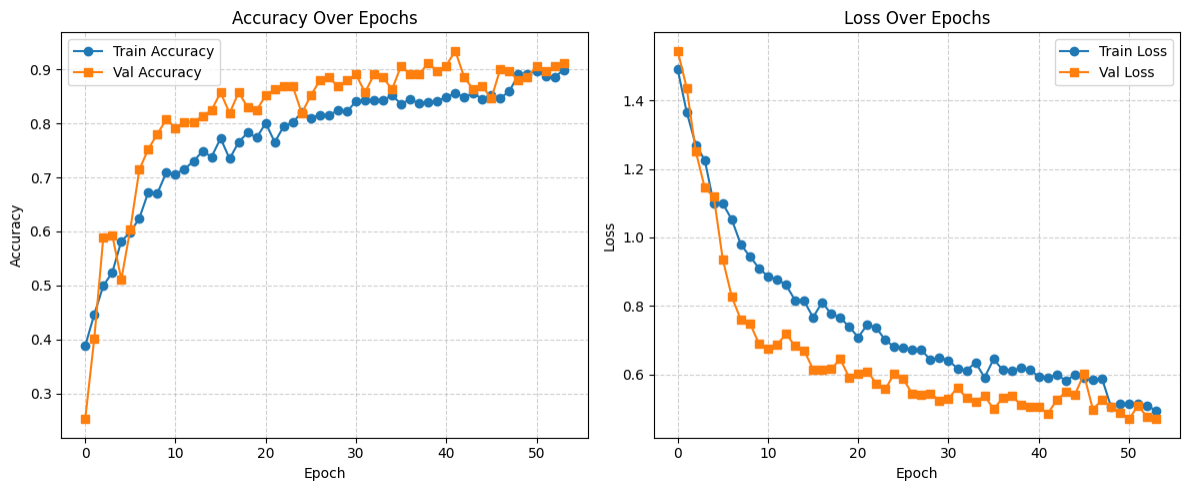
Training and validation learning curves of the proposed hybrid model. The curves show the evolution of model accuracy and loss during training.

The training process of the proposed hybrid CNN–BiLSTM–attention framework was monitored using training and validation accuracy and loss curves. As shown in Figure 4, the model showed stable convergence, with training accuracy increasing progressively and validation accuracy remaining consistently high. The validation accuracy was sometimes slightly higher than the training accuracy, which can be explained by the use of dropout and data augmentation during training. The learning curves indicate stable optimisation under the adopted learning rate scheduling, label smoothing, dropout regularisation, and early stopping strategy.

### 4.2. Validation and Held-Out Recording-Level Test Performance

The model was first evaluated on the validation set and then on the isolated held-out recording-level test set. Table 5 reports the representative single-run performance used for detailed confusion matrix, ROC, precision–recall, and XAI analyses. The validation set contained 182 recordings, and the held-out test set also contained 182 recordings.

**Figure 5:**
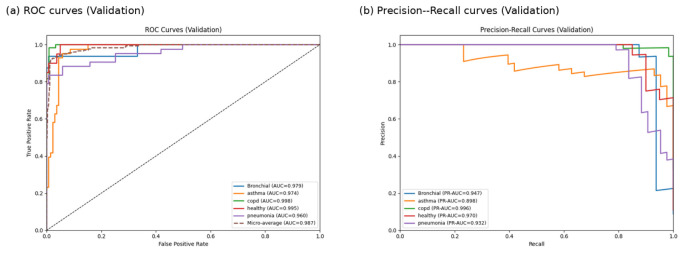
Validation-set performance analysis of the proposed hybrid model using ROC and precision–recall curves.

**Figure 6:**
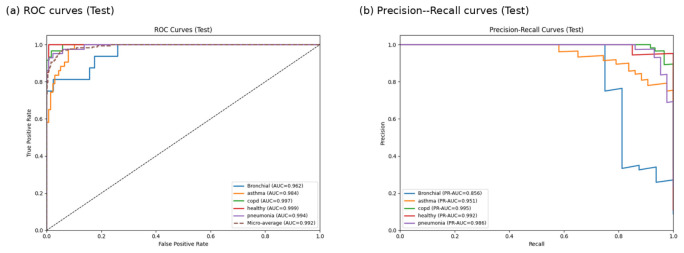
Representative single-run held-out recording-level test performance analysis of the proposed hybrid model using ROC and precision–recall curves.

In the representative run, the proposed hybrid model achieved 0.9231 validation accuracy and 0.9231 held-out recording-level test accuracy. On the held-out test set, the model achieved 0.9008 balanced accuracy, 0.9180 macro precision, 0.9008 macro recall, 0.9074 macro F1-score, 0.9842 macro ROC–AUC, and 0.9496 macro PR–AUC. The validation ROC and precision–recall curves are shown in Figure 5, while the corresponding held-out recording-level test curves are shown in Figure 6. These results indicate strong internal recording-level discrimination in the selected run. However, the repeated-run analysis in Table 6 provides the primary estimate of model stability across random seeds.

### 4.3. Confusion Matrix Analysis

**Figure 7:**
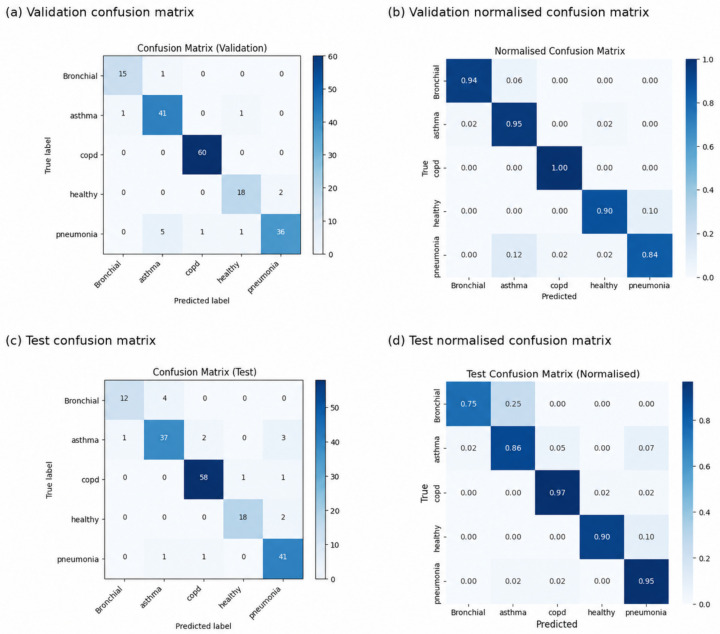
Representative single-run confusion matrix analysis of the proposed hybrid model. The figure presents validation and held-out recording-level test confusion matrices using both raw counts and normalised values.

Confusion matrix analysis was used to examine class-wise prediction behaviour in the representative run. As shown in Figure 7, both validation and held-out recording-level test confusion matrices show strong diagonal dominance. The model correctly classified 168 out of 182 validation recordings and 168 out of 182 held-out test recordings, corresponding to 92.31% accuracy in both sets. The remaining errors likely reflect acoustic overlap among respiratory conditions that may share wheezing, airflow limitation, or abnormal breath-sound characteristics. Therefore, the confusion matrices should be interpreted as descriptive class-wise evidence for the representative run, while the repeated-seed results provide the main estimate of performance stability.

### 4.4. Repeated-Run Stability Analysis

To assess stability beyond a single run, the full hybrid model was trained and evaluated across five random seeds: 42, 101, 202, 303, and 404. Results are reported as mean ± standard deviation in Table 6. Across the five repeated runs, the model achieved a mean held-out recording-level test accuracy of 0.9099±0.0163, balanced accuracy of 0.8936±0.0152, macro F1-score of 0.8937±0.0177, macro ROC–AUC of 0.9867±0.0010, and macro PR–AUC of 0.9489±0.0044. These results support stable internal recording-level performance, but they should not be interpreted as verified at patient level or clinically generalisable.

Because five repeated runs were performed, approximate 95% confidence intervals were computed as $\bar{x}\pm t_{0.975,4}\left(s/\sqrt{5}\right)$, where $\bar{x}$ is the mean, *s* is the standard deviation, and $t_{0.975,4}=2.776$. The resulting confidence intervals for validation and held-out recording-level test performance are reported in Table 7.

### 4.5. Probability Calibration Analysis

**Figure 8:**
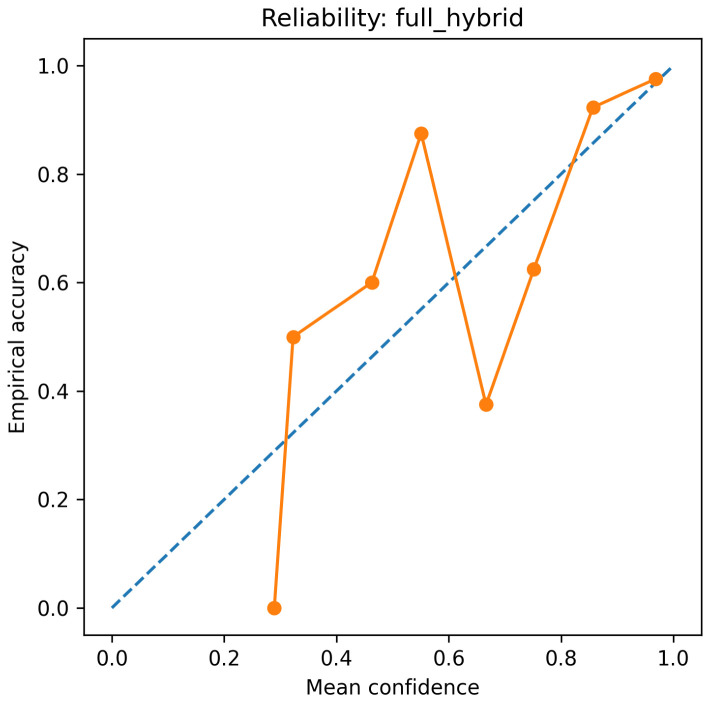
Reliability curve of the Full Hybrid model on the main held-out recording-level test split. The orange model calibration curve shows the relationship between mean predicted confidence and empirical accuracy, while the blue dashed diagonal line represents perfect calibration.

Because ROC–AUC and PR–AUC evaluate ranking performance rather than the reliability of predicted probabilities, probability calibration was assessed using expected calibration error (ECE), Brier score, negative log likelihood (NLL), and reliability curves. On the main held-out recording-level test split, the Full Hybrid model achieved an ECE of 0.054, Brier score of 0.156, and NLL of 0.321. The No-Attention Hybrid model achieved an ECE of 0.050, Brier score of 0.122, and NLL of 0.258, while the CNN–BiLSTM Mel model achieved an ECE of 0.067, Brier score of 0.161, and NLL of 0.343. The probability calibration metrics are reported in Table 8, and the reliability curve of the Full Hybrid model is shown in Figure 8.

These results indicate moderate internal probability calibration under the recording-level protocol. However, calibration was assessed only under internal held-out recording-level evaluation. Therefore, predicted probabilities and softmax outputs should not be interpreted as clinically actionable risk estimates without prospective calibration, verified patient-level testing, and clinical evaluation.

### 4.6. Exploratory Sensitivity Analysis for Data-Quality and Split Robustness

Exploratory sensitivity analyses were conducted using a separate seed 42 retraining audit to examine whether the internal recording-level results were sensitive to dataset quality and split-related issues. These analyses evaluated the Full Hybrid model under duplicate-removed, clipped-removed, duplicate-plus-clipped-removed, filename identifier-aware, and strict identifier-aware protocols. The purpose was not to replace the main five-seed repeated-run evaluation, but to assess whether exact duplicate audio, clipped recordings, and filename-derived identifier overlap could influence internal performance estimates. The results of these exploratory sensitivity analyses are summarised in Table 9.

The duplicate-removed, clipped-removed, and duplicate-plus-clipped-removed settings produced performance broadly comparable to the all-recordings sensitivity rerun, suggesting that the model did not rely solely on exact duplicate audio or clipped recordings. However, the filename identifier-aware and strict identifier-aware protocols produced substantially lower performance. In the strict identifier-aware protocol, accuracy decreased to 0.7136, balanced accuracy decreased to 0.6930, and macro F1-score decreased to 0.6822. This indicates that subject-like or source-level overlap may inflate internal performance estimates when only recording-level splitting is used. Therefore, the main results should be interpreted as held-out recording-level performance, while verified patient-level evaluation remains necessary before stronger generalisation or clinical claims can be made.

### 4.7. Comparison with Conventional Machine-Learning Baselines

Conventional machine-learning baselines were trained using handcrafted acoustic features and evaluated on the same held-out recording-level test partition. As shown in Table 10, the proposed hybrid model achieved higher mean accuracy, balanced accuracy, macro recall, macro F1-score, and macro ROC–AUC than Logistic Regression, SVM-RBF, and Random Forest. Random Forest remained competitive, particularly for macro precision and macro PR–AUC, showing that handcrafted feature-based classifiers can still provide useful threshold-independent ranking performance.

Compared with Random Forest, the proposed model improved mean test accuracy by approximately 2.53 percentage points and macro F1-score by approximately 1.80 percentage points. However, Random Forest achieved a slightly higher macro PR–AUC. Therefore, the comparison supports the value of deep spectro-temporal representation learning while also showing that handcrafted acoustic features remain useful for interpretable respiratory sound modelling.

### 4.8. Ablation Study Results

The ablation study evaluated five architectural variants: Full Hybrid, Deep-Only, Handcrafted-Only, CNN-Only, and No-Attention. The results on the held-out recording-level test set are shown in Table 11. This was a separate single-run architectural comparison and should not be interpreted as replacing the five-seed repeated-run summary in Table 6.

The Deep-Only Model achieved the highest accuracy and macro F1-score, indicating that the mel-spectrogram CNN–BiLSTM pathway was the main contributor to classification performance. The No-Attention Model achieved the highest balanced accuracy, macro ROC–AUC, and macro PR–AUC, suggesting that the attention mechanism did not consistently improve all quantitative metrics in this setting. The Full Hybrid Model should therefore not be interpreted as universally superior across all metrics. Its value lies in combining competitive internal performance with interpretable handcrafted acoustic descriptors and complementary multimodal explainability.

### 4.9. External Domain-Shift Evaluation

Domain-shift evaluation was conducted without retraining or fine tuning using two external respiratory audio resources: the ICBHI Respiratory Sound Database [31] and RespiratoryDatabase@TR [32]. Because these datasets differ from the internal dataset in recording conditions, diagnostic-label structure, population characteristics, and class distribution, these experiments were treated as domain-shift evaluations rather than evidence of readiness for clinical use.

#### 4.9.1. ICBHI Healthy-Versus-Disease Evaluation

The primary ICBHI evaluation mapped Healthy recordings to the Healthy category and all non-Healthy ICBHI labels to a broad Disease category. Table 12 summarises the recording-level and patient-level Healthy-versus-Disease results across the five repeated-seed models.

Although recording-level accuracy appears high, balanced accuracy was 0.5000 and Healthy specificity was 0.0000. This means that the model failed to identify Healthy cases in this external setting. Therefore, the ICBHI Healthy-versus-Disease results indicate limited transfer under dataset shift, particularly for Healthy-case recognition.

#### 4.9.2. ICBHI Overlapping Four-Class Evaluation

A secondary ICBHI evaluation was performed using the overlapping labels Asthma, COPD, Healthy, and Pneumonia. Table 13 summarises the four-class results.

The four-class ICBHI evaluation showed a large gap between accuracy and class-balanced metrics. Although recording-level accuracy was relatively high, balanced accuracy and macro F1-score were low at both recording and patient levels. These findings support the need for harmonised external datasets, domain adaptation, and verified patient-level evaluation.

#### 4.9.3. COPD-Focused Domain-Shift Evaluation

RespiratoryDatabase@TR was used as a COPD-focused domain-shift cohort. Because this dataset provides COPD severity labels rather than the same five internal diagnostic categories, it was not treated as a full five-class external test. The analysis focused on COPD prediction rate, mean COPD probability, prediction distribution, and the association between predicted COPD probability and COPD severity stage. The COPD-focused domain-shift evaluation results are summarised in Table 14.

The COPD-focused results showed seed-dependent COPD recognition. The weak Spearman correlation and non-significant Kruskal–Wallis trend indicate that the model should not be interpreted as predicting COPD severity. These findings reinforce that strong internal recording-level performance did not translate into robust external severity modelling.

### 4.10. Explainability Findings

**Figure 9:**
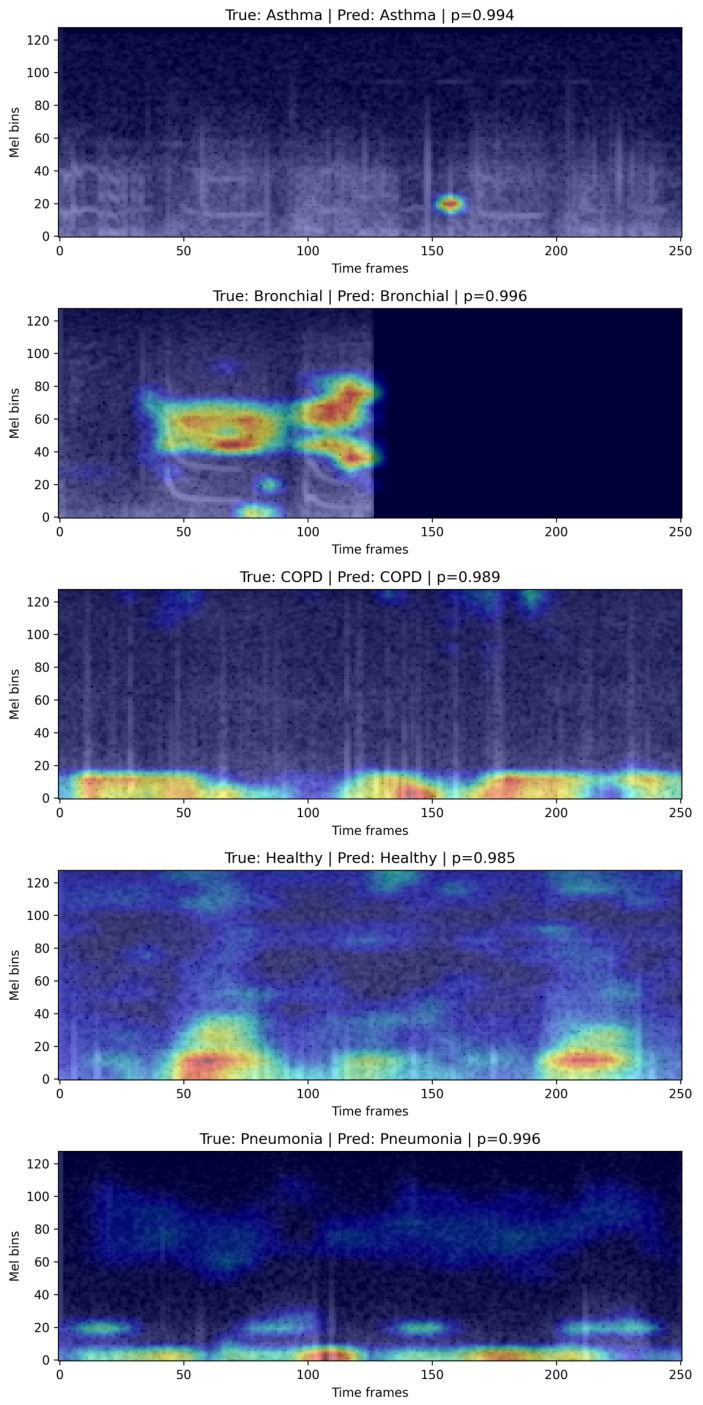
Grad-CAM visualisations for selected correctly classified held-out recording-level test examples across the five internal target classes. The heatmaps highlight spectro-temporal regions of the mel-spectrogram that contributed to the CNN-based branch predictions.

**Figure 10:**
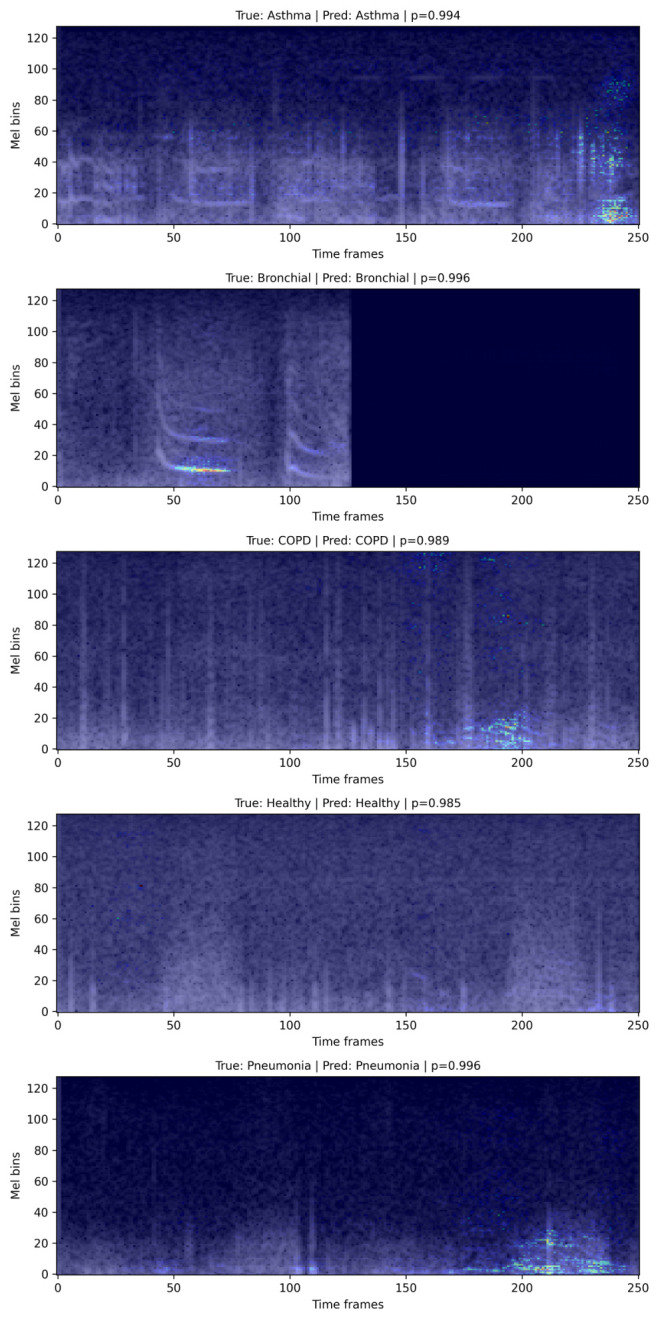
Integrated Gradients attribution maps for selected correctly classified held-out recording-level test examples. The highlighted regions indicate mel-spectrogram areas that contributed to the predicted class probability.

**Figure 11:**
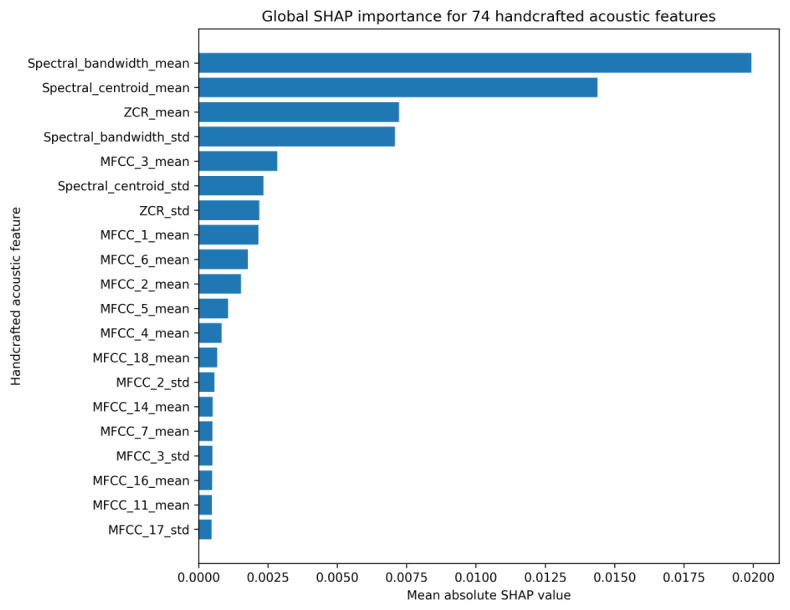
Global SHAP feature-importance analysis for the corrected 74-dimensional handcrafted acoustic-feature representation. Mean absolute SHAP values indicate the relative contribution of handcrafted descriptors to model predictions. Spectral bandwidth, spectral centroid, zero-crossing rate, and selected MFCC features contributed most strongly in the handcrafted-feature branch.

The proposed framework used complementary explainability methods for the two model branches. Grad-CAM and Integrated Gradients were applied to the mel-spectrogram branch, while SHAP was applied to the corrected 74-dimensional handcrafted acoustic-feature branch. Therefore, the interpretability analysis was separated into spectro-temporal attribution and feature-level attribution, as shown in Figure 9, Figure 10, and Figure 11, respectively.

Grad-CAM and Integrated Gradients highlighted localised spectro-temporal regions that influenced the mel-spectrogram branch, while SHAP identified spectral bandwidth, spectral centroid, zero-crossing rate, and selected MFCC features as important handcrafted descriptors. These findings provide model behaviour evidence from both branches. However, the explanation outputs were not independently validated by respiratory clinicians and should not be interpreted as clinically confirmed biomarkers.

### 4.11. Discussion

The proposed explainable hybrid framework demonstrated stable internal recording-level performance for five-class respiratory audio classification. Across five repeated runs, the model achieved a mean held-out test accuracy of 0.9099±0.0163, macro F1-score of 0.8937±0.0177, macro ROC–AUC of 0.9867±0.0010, and macro PR–AUC of 0.9489±0.0044. These results suggest that the model learned discriminative patterns from the internal dataset, but they should be interpreted as recording-level performance because verified patient-level separation could not be confirmed.

The baseline and ablation results show that deep spectro-temporal modelling was the main contributor to classification performance. The proposed model outperformed conventional handcrafted-feature baselines in most metrics, although Random Forest remained competitive for macro PR–AUC. The ablation study further showed that the Deep-Only and No-Attention variants performed strongly, indicating that handcrafted fusion and attention did not consistently improve every metric. Therefore, the Full Hybrid model is best interpreted as a competitive and interpretable multimodal framework rather than a universally superior architecture.

The sensitivity analysis provides an important caution. When stricter filename-derived identifier grouping was used, model performance dropped substantially. In the strict identifier-aware protocol, accuracy decreased to 0.7136 and macro F1-score decreased to 0.6822. This suggests that subject-like or source-level overlap may inflate internal estimates under recording-level splitting. The main results should therefore be framed as internal held-out recording-level performance, not verified patient-level generalisation.

The calibration analysis also shows that discrimination metrics should not be treated as clinical risk estimates. Although the Full Hybrid model achieved moderate internal calibration, with an ECE of 0.054 and Brier score of 0.156, these values were obtained only under internal recording-level evaluation. Predicted probabilities therefore require prospective calibration and verified patient-level testing before they can be considered clinically meaningful.

The domain-shift evaluations revealed limited external transfer. On ICBHI Healthy-versus-Disease evaluation, the model achieved high apparent recording-level accuracy but failed to identify Healthy cases, producing a balanced accuracy of 0.5000 and Healthy specificity of 0.0000. The overlapping four-class ICBHI evaluation also produced low balanced accuracy and macro F1-score despite relatively high accuracy. The COPD-focused dataset showed seed-dependent COPD recognition and did not support COPD severity prediction. These findings show that strong internal recording-level performance does not directly translate into robust cross-dataset generalisation.

The XAI results provide useful transparency but should be interpreted cautiously. Grad-CAM and Integrated Gradients identified model-relevant spectro-temporal regions, while SHAP highlighted important handcrafted acoustic descriptors. These analyses help explain model behaviour, but they do not prove that the model learned clinically validated respiratory biomarkers. Expert review and prospective clinical testing are required before clinical interpretation of these explanations.

### 4.12. Limitations and Future Directions

This study has several limitations. First, the model was developed using a single public respiratory audio dataset; therefore, its generalisability to other cohorts, recording devices, clinical environments, and population groups remains limited. Although domain-shift evaluations were added using ICBHI and a COPD-focused dataset, these evaluations revealed substantial transfer limitations.

Second, the internal dataset was partitioned using a stratified recording-level split rather than a verified patient-level split. Filename-derived subject-like identifiers could not be confirmed as globally unique patient identifiers because some identifiers were reused across disease folders. Therefore, the reported internal results should be interpreted as held-out recording-level performance rather than verified patient-independent performance.

Third, the quality control audit identified 171 recordings with clipped samples exceeding 1% and 12 exact duplicate audio pairs. These recordings were retained to preserve the original public dataset composition, but they may affect model learning and performance estimation. The sensitivity analysis showed that duplicate-removed and clipped-removed settings remained broadly comparable to the all-recordings sensitivity rerun, whereas identifier-aware protocols produced lower performance.

Fourth, the dataset is class-imbalanced, with relatively smaller support for the Bronchial and Healthy categories. Although balanced accuracy, macro-averaged metrics, and precision–recall analysis were reported, smaller class sizes may still affect the reliability of class-wise estimates.

Fifth, the dataset contains Bronchial, Asthma, COPD, Healthy, and Pneumonia categories only. It does not contain confirmed pulmonary malignancy, neoplastic consolidation, or oncology-specific labels. Therefore, no claim can be made regarding lung cancer detection, neoplastic lesion identification, or oncology screening.

Sixth, probability calibration was assessed only under internal recording-level evaluation. Although ECE, Brier score, NLL, and reliability curves were reported, the predicted probabilities were not prospectively calibrated for clinical decision-making. Therefore, model probabilities should not be interpreted as clinically actionable risk estimates.

Seventh, although Grad-CAM, Integrated Gradients, and SHAP were used to improve transparency, the explanation outputs were not independently validated by respiratory clinicians. Therefore, the XAI results should be interpreted as post hoc model behaviour analyses rather than clinically confirmed biomarkers. Finally, the current evaluation remains retrospective and algorithmic and does not establish readiness for deployable clinical decision support.

Future research should include larger multi-center respiratory audio datasets, verified patient-level partitioning, duplicate-aware split generation or duplicate removal where appropriate, harmonised domain-shift evaluation, prospective clinical testing, and clinician-centered usability assessment. Additional work should explore improved imbalance-aware training, probability calibration, formal statistical comparison across repeated model variants, domain adaptation for external datasets, and integration with clinical metadata where ethically and practically available.

## 5. Conclusions

This study presented an explainable multimodal deep-learning framework for recording-level five-class respiratory audio classification. The model combined a mel-spectrogram-based CNN–BiLSTM–attention branch with a handcrafted acoustic-feature branch to classify Bronchial, Asthma, COPD, Healthy, and Pneumonia recordings while supporting both spectro-temporal and feature-level interpretability.

Across five repeated experiments with different random seeds, the proposed hybrid model achieved a mean held-out recording-level test accuracy of 0.9099±0.0163, balanced accuracy of 0.8936±0.0152, macro F1-score of 0.8937±0.0177, macro ROC–AUC of 0.9867±0.0010, and macro PR–AUC of 0.9489±0.0044. Compared with conventional machine learning baselines, the model achieved stronger internal performance for several metrics, including accuracy, balanced accuracy, macro recall, macro F1-score, and macro ROC–AUC. However, Random Forest remained competitive in macro precision and macro PR–AUC. Therefore, the proposed framework should be interpreted as a competitive internal recording-level classifier with multimodal interpretability rather than as a universally superior model across all metrics.

The ablation analysis showed that the deep spectro-temporal branch was the main contributor to classification performance, while the handcrafted branch provided complementary acoustic interpretability. Grad-CAM and Integrated Gradients highlighted model-relevant spectro-temporal regions in the mel-spectrogram branch, whereas SHAP identified influential handcrafted acoustic descriptors in the 74-dimensional handcrafted-feature branch. These explanation outputs improve transparency but should be interpreted as post hoc model behaviour evidence rather than clinically validated respiratory biomarkers.

Identifier-aware sensitivity analyses showed a clear reduction in performance compared with the main recording-level split. In the strict identifier-aware protocol, accuracy decreased to 0.7136, balanced accuracy decreased to 0.6930, and macro F1-score decreased to 0.6822. This finding suggests that subject-like or source-level overlap may inflate internal performance estimates when only recording-level splitting is used. Therefore, verified patient-level evaluation is required before stronger generalisation claims can be made.

Domain-shift evaluation on the ICBHI Respiratory Sound Database and a COPD-focused respiratory dataset revealed important transfer limitations. The model showed poor Healthy-case recognition on ICBHI and seed-dependent COPD recognition in the COPD-focused cohort. These findings demonstrate that strong internal held-out recording-level performance does not directly translate into robust external or clinical generalisation.

Despite the promising internal results, the study remains limited by single-dataset development, recording-level partitioning due to unavailable reliable patient identifiers, class imbalance, clipped and duplicate recordings, limited external transfer, and probability calibration restricted to internal recording-level evaluation. Although ECE, Brier score, NLL, and reliability curves were reported, predicted probabilities should not be interpreted as clinically actionable risk estimates without prospective calibration and verified patient-level testing. Importantly, the dataset does not include confirmed pulmonary malignancy, neoplastic consolidation, or oncology-specific labels; therefore, no claim is made regarding lung cancer detection, neoplastic lesion identification, or oncology screening.

Future work should focus on larger multi-center respiratory audio datasets, verified patient-level partitioning, duplicate-aware and clipping-aware evaluation, harmonised domain-shift evaluation, domain adaptation, probability calibration, prospective clinical testing, and clinician-centered assessment before real-world deployment.

## Figures and Tables

**Table 1:** Class-wise distribution of the Asthma Detection Dataset Version 2.

Class	Asthma	Bronchial	COPD	Healthy	Pneumonia	Total
Samples	288	104	401	133	285	1211
Percentage (%)	23.78	8.59	33.11	10.98	23.53	100.00

**Table 2:** Quality control audit of the internal respiratory audio dataset.

Audit Item	Operational Definition	Result
Unreadable files	WAV file could not be opened or decoded	0
Audio channels	Number of channels in each WAV file	All mono
Original sampling rate	Sampling rate before resampling	707 files at 44.1 kHz; 504 files at 4 kHz
Duration range	Original duration before trimming/padding	0.48–6.00 s
Very short recordings	Duration < 1.5 s	3 files
Shorter than model input	Duration < 4.0 s	10 files
Near-silent recordings	RMS amplitude < 0.001	0 files
Low-peak recordings	Peak amplitude < 0.01	0 files
Excessive clipping	Clipped samples > 1%	171 files
Clipped recordings by split	Train/validation/test distribution	127/21/23 files
Exact duplicate audio	Identical audio file hash	12 duplicate pairs
Final included recordings	Recordings retained for model development	1211 files

**Table 3:** Training, validation, and held-out recording-level test split distribution.

Class	Training	Validation	Held-Out Test	Total
Bronchial	72	16	16	104
Asthma	202	43	43	288
COPD	281	60	60	401
Healthy	93	20	20	133
Pneumonia	199	43	43	285
Total	847	182	182	1211

**Table 4:** Summary of the final training configuration and hyperparameters.

Parameter	Configuration
Optimiser	AdamW
Initial learning rate	$\eta_{0}=3\times 10^{-4}$
Weight decay	$1\times 10^{-5}$
Learning-rate scheduler	ReduceLROnPlateau monitoring validation loss
Scheduler factor and patience	Factor 0.5, patience 6 epochs, minimum learning rate $1\times 10^{-6}$
Batch size	16
Maximum epochs	100
Early stopping	Patience 12 epochs
Loss function	Sparse categorical cross-entropy with label smoothing
Label smoothing	ϵ=0.05
Regularisation	Dropout 0.3–0.4 and AdamW weight decay
Mixed precision	Not enabled in the final main experiment
Class weighting in main deep model	Not applied
Data split	70% training/15% validation/15% held-out test, stratified recording-level split
Repeated-run seeds	42, 101, 202, 303, and 404
Evaluation metrics	Accuracy, balanced accuracy, macro precision, macro recall, macro F1, macro ROC–AUC, and macro PR–AUC

**Table 5:** Representative single-run performance of the proposed hybrid model on the validation and held-out recording-level test sets.

Metric	Validation Set	Held-Out Recording-Level Test Set
Accuracy	0.9231	0.9231
Balanced Accuracy	0.9230	0.9008
Macro Precision	0.9243	0.9180
Macro Recall	0.9230	0.9008
Macro F1-score	0.9197	0.9074
Macro ROC–AUC	0.9795	0.9842
Macro PR–AUC	0.9408	0.9496

**Table 6:** Repeated-run stability analysis of the proposed Full Hybrid model across five random seeds. Results are reported as mean ± standard deviation.

Metric	Validation Set	Held-Out Recording-Level Test Set
Accuracy	0.9198±0.0148	0.9099±0.0163
Balanced Accuracy	0.9155±0.0159	0.8936±0.0152
Macro Precision	0.9104±0.0157	0.9015±0.0199
Macro Recall	0.9155±0.0159	0.8936±0.0152
Macro F1-score	0.9089±0.0171	0.8937±0.0177
Macro ROC–AUC	0.9820±0.0019	0.9867±0.0010
Macro PR–AUC	0.9458±0.0050	0.9489±0.0044

**Table 7:** Approximate 95% confidence intervals for repeated-run performance across five random seeds.

Metric	Validation Set 95% CI	Held-Out Recording-Level Test Set 95% CI
Accuracy	0.9014–0.9382	0.8897–0.9301
Balanced Accuracy	0.8958–0.9352	0.8747–0.9125
Macro Precision	0.8909–0.9299	0.8768–0.9262
Macro Recall	0.8958–0.9352	0.8747–0.9125
Macro F1-score	0.8877–0.9301	0.8717–0.9157
Macro ROC–AUC	0.9796–0.9844	0.9855–0.9879
Macro PR–AUC	0.9396–0.9520	0.9434–0.9544

**Table 8:** Probability calibration metrics on the main held-out recording-level test split.

Model	ECE	Brier Score	NLL
Full Hybrid	0.054	0.156	0.321
No-Attention Hybrid	0.050	0.122	0.258
CNN–BiLSTM Mel	0.067	0.161	0.343

**Table 9:** Exploratory sensitivity analysis for data quality and split robustness conditions using the Full Hybrid model with seed 42. Results are from a separate sensitivity retraining audit and should not be interpreted as replacements for the main five-seed repeated-run results.

Evaluation Protocol	Train	Val	Test	Accuracy	Balanced Accuracy	Macro F1	Macro ROC–AUC	Macro PR–AUC
All recordings: sensitivity rerun	847	182	182	0.8736	0.8630	0.8574	0.9846	0.9416
Duplicate-removed split	839	180	180	0.8778	0.8352	0.8455	0.9872	0.9414
Clipped-removed split	728	156	156	0.8782	0.8731	0.8698	0.9818	0.9531
Duplicate + clipped removed split	719	155	155	0.8968	0.8930	0.8929	0.9833	0.9434
Filename-identifier-aware split	765	202	244	0.7828	0.7758	0.7596	0.9533	0.8138
Strict identifier-aware protocol	631	192	206	0.7136	0.6930	0.6822	0.9212	0.7848

**Table 10:** Comparison between the proposed hybrid model and conventional machine learning baselines on the held-out recording-level test set.

Model	Accuracy	Balanced Accuracy	Macro Precision	Macro Recall	Macro F1	Macro ROC–AUC	Macro PR–AUC
Logistic Regression	0.8681	0.8634	0.8335	0.8634	0.8455	0.9705	0.9070
SVM-RBF	0.8626	0.8509	0.8563	0.8509	0.8496	0.9831	0.9437
Random Forest	0.8846	0.8643	0.9003	0.8643	0.8757	0.9845	**0.9510**
**Proposed Hybrid Model**	0.9099±0.0163	0.8936±0.0152	0.9015±0.0199	0.8936±0.0152	0.8937±0.0177	0.9867±0.0010	0.9489±0.0044

*Note:* Conventional machine learning baselines are reported from the representative experimental setting, whereas the proposed hybrid model is reported as mean ± standard deviation across five repeated runs. Bold values indicate the strongest point estimate for each metric. Comparisons should be interpreted descriptively because repeated-run variability is reported only for the proposed hybrid model.

**Table 11:** Ablation study results on the held-out recording-level test set. The results represent a separate single-run architectural comparison and should not be interpreted as replacements for the five-seed repeated-run performance summary.

Model Variant	Accuracy	Balanced Accuracy	Macro Precision	Macro Recall	Macro F1	Macro ROC–AUC	Macro PR–AUC
Full Hybrid Model	0.8956	0.8774	0.8971	0.8774	0.8845	0.9824	0.9369
Deep-Only Model	**0.9121**	0.8834	**0.9345**	0.8834	**0.9034**	0.9856	0.9469
Handcrafted-Only Model	0.8571	0.8238	0.8714	0.8238	0.8371	0.9641	0.8954
CNN-Only Model	0.8132	0.7838	0.8358	0.7838	0.7968	0.9708	0.8880
No-Attention Model	0.8956	**0.8855**	0.8830	**0.8855**	0.8779	**0.9886**	**0.9535**

**Table 12:** ICBHI Healthy-versus-Disease domain-shift evaluation across five repeated-seed models. Results are reported as mean ± standard deviation.

Metric	Recording Level	Patient Level
Accuracy	0.9620±0.0000	0.7937±0.0000
Balanced Accuracy	0.5000±0.0000	0.5000±0.0000
Macro F1-score	0.4903±0.0000	0.4425±0.0000
Disease Sensitivity	1.0000±0.0000	1.0000±0.0000
Healthy Specificity	0.0000±0.0000	0.0000±0.0000

**Table 13:** ICBHI overlapping four-class domain-shift evaluation across five repeated-seed models. Results are reported as mean ± standard deviation.

Metric	Recording Level	Patient Level
Accuracy	0.8804±0.0489	0.6454±0.0201
Balanced Accuracy	0.3045±0.0966	0.3013±0.1008
Macro F1-score	0.2495±0.0267	0.2157±0.0235

**Table 14:** COPD-focused domain-shift evaluation on RespiratoryDatabase@TR across five repeated-seed models. Results are reported as mean ± standard deviation.

Metric	Result
COPD prediction rate	0.7667±0.3284
Mean COPD probability	0.7521±0.3059
Spearman correlation between COPD stage and COPD probability	0.1406±0.1061
Kruskal–Wallis *p*-value for COPD probability across stages	0.4403±0.3170

## Data Availability

The source code used in this study is publicly available in the GitHub repository: https://github.com/Arefin-Saiful/Lung-Diseases-Detection (accessed on 20 June 2026). The main dataset used for internal model development and evaluation was the Asthma Detection Dataset Version 2, available from Kaggle: https://www.kaggle.com/datasets/mohammedtawfikmusaed/asthma-detection-dataset-version-2 (accessed on 30 June 2025). External domain-shift evaluation was conducted using two publicly available datasets: the ICBHI Respiratory Sound Database, available from Kaggle: https://www.kaggle.com/datasets/vbookshelf/respiratory-sound-database (accessed on 30 June 2025), and RespiratoryDatabase@TR, available from Mendeley Data: https://data.mendeley.com/datasets/p9z4h98s6j/1 (accessed on 10 May 2026).

## References

[1] World Health Organization (2025). Chronic Respiratory Diseases: More Than 80 Million Affected and Many More Undiagnosed, Warns New WHO and European Respiratory Society Report. https://www.who.int/europe/news/item/12-06-2025-chronic-respiratory-diseases--more-than-80-million-affected-and-many-more-undiagnosed--warns-new-who-andeuropean-respiratory-society-report.

[2] Kapetanidis P., Kalioras F., Tsakonas C., Tzamalis P., Kontogiannis G., Karamanidou T., Stavropoulos T.G., Nikoletseas S. (2024). Respiratory diseases diagnosis using audio analysis and artificial intelligence: A systematic review. Sensors.

[3] Joy R.P., Haider N.S. (2021). Classification of lung disorders based on lung sounds using deep learning. J. Phys. Conf. Ser..

[4] Kim Y., Hyon Y., Jung S.S., Lee S., Yoo G., Chung C., Ha T. (2021). Respiratory sound classification for crackles, wheezes, and rhonchi in the clinical field using deep learning. Sci. Rep..

[5] Alqudah A.M., Qazan S., Obeidat Y.M. (2022). Deep learning models for detecting respiratory pathologies from raw lung auscultation sounds. Soft Comput..

[6] Yadav S., Agarwal P., Rizvi S.A.M. (2024). Automated Multiclass Classification of Lung Diseases with Deep Learning: A Hybrid CNN–LSTM Approach. Proceedings of the 2024 4th International Conference on Technological Advancements in Computational Sciences (ICTACS).

[7] Gopi G., Varghese A. (2025). Deep Learning for Respiratory Sound-Based Disease Classification. Proceedings of the 2025 International Conference on Advancements in Power, Communication and Intelligent Systems (APCI).

[8] Sanjana J., Naik P.P., Padukudru M.A., Koolagudi S.G., Rajan J. (2023). Attention-Based CRNN Models for Identification of Respiratory Diseases from Lung Sounds. Proceedings of the 2023 14th International Conference on Computing Communication and Networking Technologies (ICCCNT).

[9] Karaarslan O., Belcastro K.D., Ergen O. (2024). Respiratory sound-base disease classification and characterization with deep/machine learning techniques. Biomed. Signal Process. Control.

[10] Shehab S.A., Mohammed K.K., Darwish A., Hassanien A.E. (2024). Deep learning and feature fusion-based lung sound recognition model to diagnoses the respiratory diseases. Soft Comput..

[11] Vasava R.P., Joshiara H.A. (2023). Different Respiratory Lung Sounds Prediction using Deep Learning. Proceedings of the 2023 4th International Conference on Electronics and Sustainable Communication Systems (ICESC).

[12] Jung S.Y., Liao C.H., Wu Y.S., Yuan S.M., Sun C.T. (2021). Efficiently classifying lung sounds through depthwise separable CNN models with fused STFT and MFCC features. Diagnostics.

[13] Wang Q., Bu Z., Mao J., Zhu W., Zhao J., Du W., Shi G., Zhou M., Chen S., Qu J. (2024). Towards reliable respiratory disease diagnosis based on cough sounds and vision transformers. arXiv.

[14] Petmezas G., Cheimariotis G.A., Stefanopoulos L., Rocha B., Paiva R.P., Katsaggelos A.K., Maglaveras N. (2022). Automated lung sound classification using a hybrid CNN–LSTM network and focal loss function. Sensors.

[15] Abhishek S., Ananthapadmanabhan A., Anjali T., Reyma S., Perathur A., Bentov R.B. (2024). Multimodal Integration of an Enhanced Novel Pulmonary Auscultation Real-Time Diagnostic System. IEEE Multimed..

[16] Zhao Z., Gong Z., Niu M., Ma J., Wang H., Zhang Z., Li Y. (2022). Automatic respiratory sound classification via multi-branch temporal convolutional network. Proceedings of the ICASSP 2022-2022 IEEE International Conference on Acoustics, Speech and Signal Processing (ICASSP).

[17] Singh A.P., Nigam A., Garg G. (2025). Evaluation of Deep Learning Methods for Pulmonary Disease Classification. Curr. Med. Imaging.

[18] Zhang Y., Huang Q., Sun W., Chen F., Lin D., Chen F. (2024). Research on lung sound classification model based on dual-channel CNN-LSTM algorithm. Biomed. Signal Process. Control.

[19] S O., Sudhagar G., M H. Deep Attention Fusion Framework on Lung Sound Classification by Multi-Spectral Features. Proceedings of the 2025 9th International Conference on Inventive Systems and Control (ICISC).

[20] Pham L., McLoughlin I., Phan H., Tran M., Nguyen T., Palaniappan R. (2020). Robust deep learning framework for predicting respiratory anomalies and diseases. Proceedings of the 2020 42nd Annual International Conference of the IEEE Engineering in Medicine & Biology Society (EMBC).

[21] Chamberlain D., Kodgule R., Ganelin D., Miglani V., Fletcher R. (2016). Application of Semi-Supervised Deep Learning to Lung Sound Analysis. Proceedings of the 2016 38th Annual International Conference of the IEEE Engineering in Medicine and Biology Society (EMBC).

[22] Perna D., Tagarelli A. (2019). Deep auscultation: Predicting respiratory anomalies and diseases via recurrent neural networks. Proceedings of the 2019 IEEE 32nd International Symposium on Computer-Based Medical Systems (CBMS).

[23] Bhushan P., Fahad M.S., Agrawal S., Kamesh K.S.D., Tripathi P., Mishra P., Singh V.K., Deepak A. (2024). A Self-Attention Based Hybrid CNN-LSTM Architecture for Respiratory Sound Classification. GMSARN Int. J..

[24] Li C., Liu S. (2025). TM2SP: A Transformer-based Multi-Level Spatiotemporal Feature Pyramid Network for Video Saliency Prediction. IEEE Trans. Circuits Syst. Video Technol..

[25] Fernando T., Sridharan S., Denman S., Ghaemmaghami H., Fookes C. (2022). Robust and interpretable temporal convolution network for event detection in lung sound recordings. IEEE J. Biomed. Health Inform..

[26] Akman A., Schuller B.W. (2024). Audio explainable artificial intelligence: A review. Intell. Comput..

[27] Lo Giudice M., Mammone N., Ieracitano C., Aguglia U., Mandic D., Morabito F.C. (2022). Explainable deep learning classification of respiratory sound for telemedicine applications. Proceedings of the International Conference on Applied Intelligence and Informatics.

[28] Saraswat D., Bhattacharya P., Verma A., Prasad V.K., Tanwar S., Sharma G., Bokoro P.N., Sharma R. (2022). Explainable AI for healthcare 5.0: Opportunities and challenges. IEEE Access.

[29] Amann J., Blasimme A., Vayena E., Frey D., Madai V.I., Consortium P. (2020). Explainability for artificial intelligence in healthcare: A multidisciplinary perspective. Bmc Med. Inform. Decis. Mak..

[30] Sun Z. (2023). ICBHI 2017 Challenge.

[31] Rocha B.M., Filos D., Mendes L., Vogiatzis I., Perantoni E., Kaimakamis E., Natsiavas P., Oliveira A., Jácome C., Marques A. (2017). A respiratory sound database for the development of automated classification. Proceedings of the International Conference on Biomedical and Health Informatics.

[32] Altan G., Kutlu Y. (2020). RespiratoryDatabase@TR (COPD Severity Analysis).

